# Underestimated Manipulative Roles of *Mycobacterium tuberculosis* Cell Envelope Glycolipids During Infection

**DOI:** 10.3389/fimmu.2019.02909

**Published:** 2019-12-18

**Authors:** Andreu Garcia-Vilanova, John Chan, Jordi B. Torrelles

**Affiliations:** ^1^Population Health Program, TB Group, Texas Biomedical Research Institute, San Antonio, TX, United States; ^2^Department of Medicine (Infectious Diseases), Albert Einstein College of Medicine & Montefiore Medical Center, Bronx, NY, United States; ^3^Department of Microbiology and Immunology, Albert Einstein College of Medicine & Montefiore Medical Center, Bronx, NY, United States

**Keywords:** tuberculosis, *Mycobacterium tuberculosis*, cell envelope glycolipids, infectious diseases, vaccine strategies, immunomodulatory lipids, immune responses

## Abstract

The *Mycobacterium tuberculosis* cell envelope has been evolving over time to make the bacterium transmissible and adaptable to the human host. In this context, the *M. tuberculosis* cell envelope contains a peripheral barrier full of lipids, some of them unique, which confer *M. tuberculosis* with a unique shield against the different host environments that the bacterium will encounter at the different stages of infection. This lipid barrier is mainly composed of glycolipids that can be characterized by three different subsets: trehalose-containing, mannose-containing, and 6-deoxy-pyranose-containing glycolipids. In this review, we explore the roles of these cell envelope glycolipids in *M. tuberculosis* virulence and pathogenesis, drug resistance, and further, how these glycolipids may dictate the *M. tuberculosis* cell envelope evolution from ancient to modern strains. Finally, we address how these *M. tuberculosis* cell envelope glycolipids are impacted by the host lung alveolar environment, their role in vaccination and masking host immunity, and subsequently the impact of these glycolipids in shaping how *M. tuberculosis* interacts with host cells, manipulating their immune response to favor the establishment of an infection.

## Introduction

Currently, in the twenty-first century, one person is infected with *Mycobacterium tuberculosis* (*M. tuberculosis*) every 6 s, and one person dies of tuberculosis (TB) every 21 s. Multi-, extensively, extremely (also known as total) drug-resistant (MDR/XDR/XXDR/TDR) *M. tuberculosis* strains are emerging worldwide as a threat to public health and TB control, raising concerns of a future epidemic of virtually untreatable TB (1–5). It is estimated that drug-resistant TB will kill 75 million people and cost the global economy $16.7 trillion over the next 35 years (6).

Most current and proposed therapies target the *M. tuberculosis* cell envelope, a complex and dynamic structure. Despite this, little is known regarding the role of *M. tuberculosis* cell envelope lipids, and specially glycolipids, in the establishment and maintenance of the infection and in the development of active TB disease. During its path to infection and disease outcome, *M. tuberculosis* gets in contact with different host environments (7), where a critical gap in our knowledge is defining the changes in the *M. tuberculosis* cell envelope structures during the course of pulmonary infection, as well as how these changes determine the *M. tuberculosis* infection outcome.

**Figure 1:**
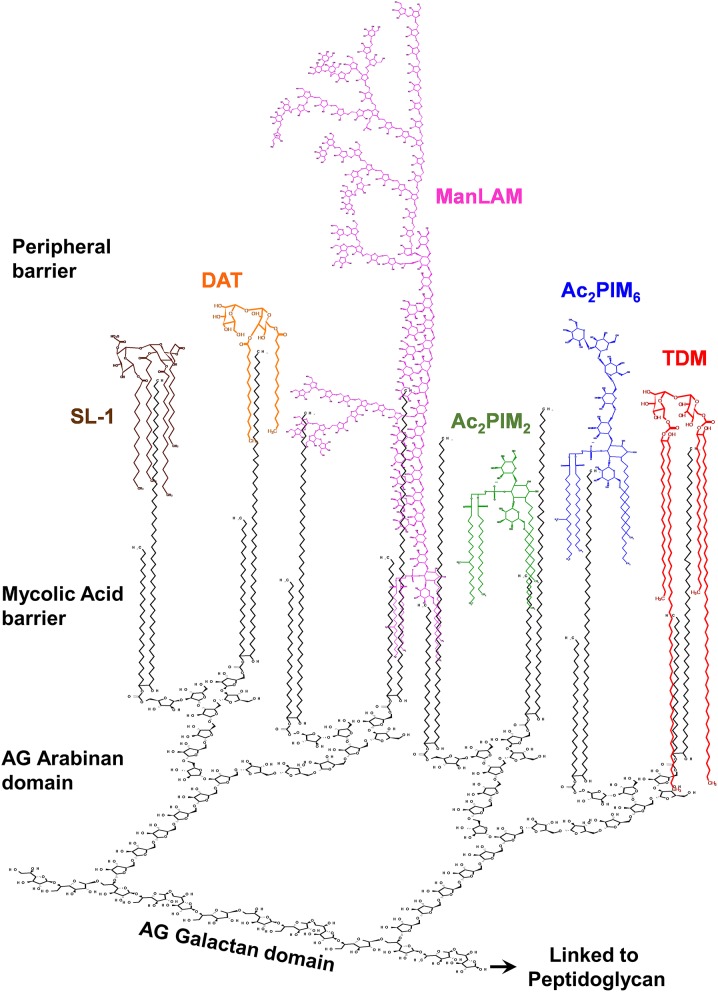
The *M. tuberculosis* complex cell envelope with emphasis on surface glycolipids. The *M. tuberculosis* cell envelope is composed of a covalently linked core called mycolic-acids-arabinogalactan-peptidoglycan (mAGP) complex. On the top of this core is described to be a peripheral lipid barrier, where lipids intercalate among the mycolic acids of mAGP. Mycolic acids are perpendicular to the plasma membrane. Here the *M. tuberculosis* cell envelope surface location of mannose-containing glycolipids (phosphatidyl-*myo*-inositol mannosides, or PIMs) and lipoglycans (e.g., the mannose capped lipoarabinomannan, or ManLAM), as well as trehalose containing lipids [e.g., trehalose-dimycolate (TDM), sulfolipid-1 (SL-1), and diacyl-trehalose (DAT)], is depicted. The trehalose containing lipooligoscharides (LOSs), phthienoates and hydroxyphthienoates, 6-deoxy-pyranose-lipids (phenolic glycolipids, or PGLs), and mannose-containing mannosyl-β-1–phosphomycoketides (MPM) are not depicted. Outer material components (i.e., α-glucan, mannan, arabinomannan, and xylan) are also not shown. Relative number of molecules and size are not accurately depicted, reflecting published experimental data.

**Figure 2:**
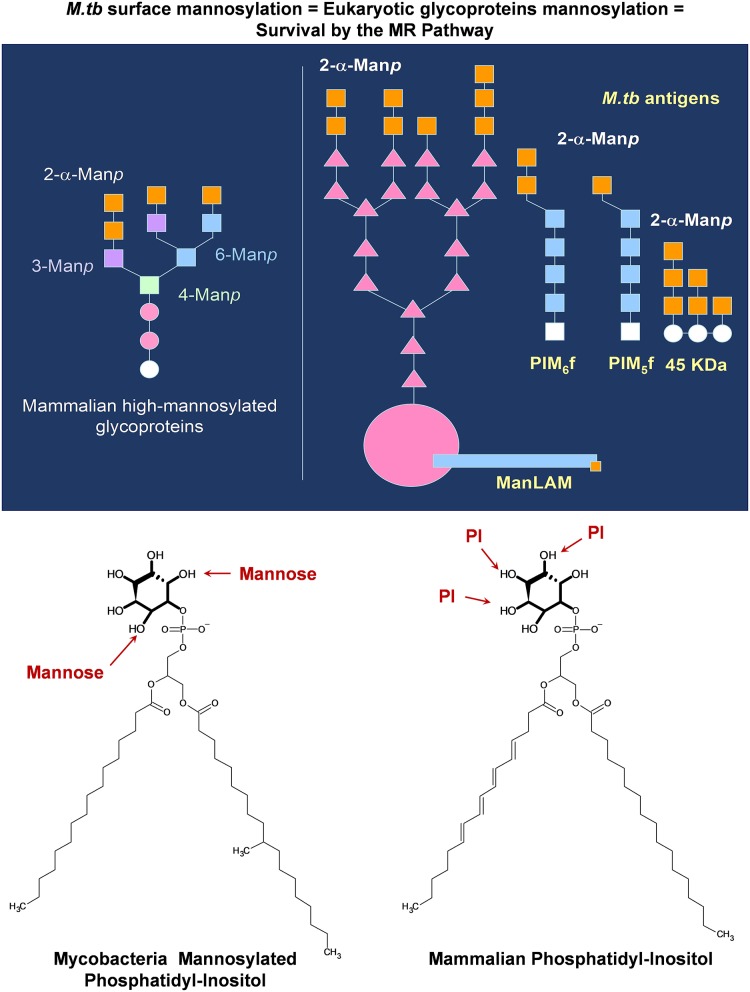
*M. tuberculosis* complex mannose-containing lipids mimic mammalian structures allowing the bacterium to gain entry into host cells and thus potentially bypassing the host immune response. *M. tuberculosis* mannose-containing glycolipids (PIMs) and lipoglycans [ManLAM and lipomannan (LM), and some mannose containing proteins] contain α-2-mannose in their non-reducing termini, mimicking non-siliated mammalian glycoproteins in circulation. These non-siliated mammalian proteins can trigger inflammation, and thus, the host contains a series of homeostatic receptors, such as the mannose receptor (MR), to remove them out from circulation. *M. tuberculosis* can take advantage of this host clearance system by increasing its mannose surface, allowing the *M. tuberculosis* bacterium to interact with the homeostatic MR (among others), gaining entry and surviving within host cells. Moreover, these same glycolipids and lipoglycans contain a GPI-anchor of similar structure to mamalian phosphatidyl-inositol, which intercalates within vesicular membranes, regulating vesicular trafficking and fusion.

Little is also known about the cell envelope composition of MDR/XDR/XXDR/TDR *M. tuberculosis* strains. For these strains, it is still unknown what bacterial and host factors are involved in the induction of their overwhelming tissue damaging, driving to the collapse of the lung merely months after active TB is diagnosed. The *M. tuberculosis* cell envelope, in its majority (~80%), comprises carbohydrates and lipids readily exposed to the host immune system and thus being instrumental in determining the *M. tuberculosis* pathway of infection and the host inflammatory response that defines the TB disease outcome. In this context, structural-biological function relationships for the majority of the *M. tuberculosis* cell envelope glycolipids are still being elucidated. Mainly, *M. tuberculosis* glycolipids are composed of two carbohydrates, trehalose (a glucose disaccharide) and mannose (Figure 1). Related to the *M. tuberculosis* cell envelope trehalose glycolipid content, it is plausible that trehalose synthesis and degradation play a role in the process of reciprocal carbon mobilization across the *M. tuberculosis* cell envelope, especially during infection, where the regulation of trehalose containing lipids on the *M. tuberculosis* cell envelope may be modulated by many environmental and physiological factors that affect both *M. tuberculosis* and the host cell (8, 9). In regard to mannose-containing glycolipids and lipoglycans, studies have focused on showing how *M. tuberculosis* mimics the host by containing cell envelope components (e.g., mannose-containing molecules) whose terminal epitopes closely resemble mammalian glycoforms such as mannoproteins (Figure 2). Indeed, studies have shown that *M. tuberculosis* uses this host resemblance to its advantage, gaining entrance and establishing a unique intracellular niche within the host (10, 11).

Many *M. tuberculosis*-host factors are involved in this first encounter interface at the initial stages of the infection. First, the constitution of the *M. tuberculosis* cell envelope, which is strain-dependent, from the worldwide used laboratory strains (*M. tuberculosis* H_37_R_a_, *M. tuberculosis* H_37_R_v_, and *M. tuberculosis* Erdman), to the highly transmissible CDC1551, to the hypervirulent HN878, and to several relevant drug-resistant *M. tuberculosis* clinical isolates in endemic TB areas (12–16). We will discuss their cell envelope glycolipid constitution in relation to their infection outcome. Second, we will focus on the alveolar space and its predominant cells, the alveolar macrophage (AM) and the alveolar epithelial cell, and how these alveolar cells act as a niche for *M. tuberculosis* and discuss their contribution to control or favor the infection. We will also discuss our results in an area frequently bypassed, the host lung alveolar environment and its impact on the *M. tuberculosis* cell envelope, addressing how the lung environment can determine and change the nature of the *M. tuberculosis* cell envelope during infection and how these changes on the *M. tuberculosis* cell envelope may determine/contribute to the pathway of infection and disease outcome. Importantly, we will also discuss our recent findings on the potential role of the mycobacterial glycolipids in masking the generation of the host protective immune response against *M. tuberculosis* infection and TB disease.

## The *M. tuberculosis* Cell Envelope and Its Glycolipids

The *M. tuberculosis* complex includes *M. tuberculosis, M. africanum, M. bovis*, and the Bacillus Calmette–Guérin (BCG) strain, *M. canetti, M. caprae, M. microti, M. mungi, M. orygis, M. pinnipedii*, and *M. suricattae* (with *M. dassie* being not yet included). The *M. tuberculosis* complex cell envelope has a unique, complex, thick, and waxy cell envelope, with properties, such as low permeability and intrinsic resistance to entry of many hydrophobic antibiotics and resistance to harsh environments which, distinguish *M. tuberculosis* from other pathogenic bacteria (17, 18). This envelope comprises an array of lipids with diverse polarity, such as glycolipids, aminolipids, phospholipids, phosphoglycolipids, sulfolipids, phenolic-glycolipids, and other lipids; all of them exhibit important roles in *M. tuberculosis* pathogenicity and are an important target, due to their alleged essentiality and proved immunogenicity, for new drug treatments and diagnostics. Most, if not all, *M. tuberculosis* cell envelope components have been shown to provide *M. tuberculosis* with some advantage for establishing an infection or survival in the host, for inhibiting bacteriostatic components of the host lung mucosa, or by allowing it to survive harsh intracellular environments, adapting its metabolism to the host environment. Thus, the cell envelope of *M. tuberculosis, per se*, may contain all of the elements associated with active TB disease, including elements driving tissue caseation, hypersensitization, cytotoxicity, and the antigens triggering or dampening humoral and adaptive immunity (19). However, although obvious, cell envelope compositional differences exist among *M. tuberculosis* complex strains and non-pathogenic mycobacteria; a detailed electron microscopy study has not yet identified any special and unique features on the *M. tuberculosis* complex strains.

The *M. tuberculosis* cell envelope is defined in five distinct compartments, the plasma membrane, a periplasmic space, the cell envelope core, the peripheral lipid layer, and around it, the outer material. All these parts are thought to provide structural, mechanical, and metabolic support, as well as osmotic protection and transport and communication with host environments surrounding the bacillus during different stages of infection. Here, we will discuss *M. tuberculosis* cell envelope, focusing on the *M. tuberculosis* peripheral lipid layer constituents and their role in *M. tuberculosis* pathogenesis.

## The *M. tuberculosis* Cell Envelope Peripheral Lipid Layer

**Figure 3:**
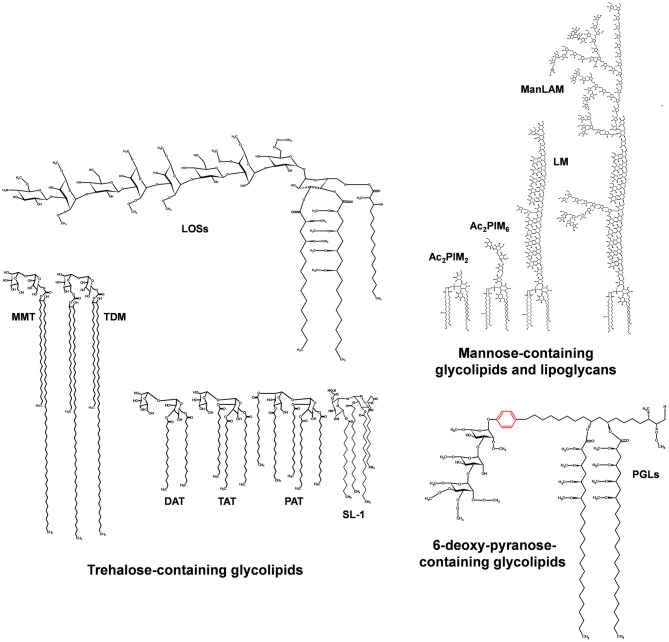
Major glycolipids of the *M. tuberculosis* cell envelope surface. Three groups of glycolipids can be found on the *M. tuberculosis* cell envelope: (i) trehalose containing lipids, which involve di-, tri-, and penta-acylated trehaloses (DAT, TAT, PAT); trehalose mono- and di-mycolate (TMM, TDM); sulfolipids (SL-1); and lipooligosaccharides (LOSs); (ii) mannose-containing lipids, which involve the phosphatidyl-*myo*-inositol mannosides of different lengths (1 to 6 mannoses) and acylation patterns 1 to 4 fatty acid of different nature, being the most common palmitic (16:0) and tuberculostearic acid (TBST); and (iii) 6-deoxy-pyranose containing lipids, mainly phenolic glycolipids (PGLs). LOS and PGL types are restricted to a few strains of the *M. tuberculosis* complex.

**Figure 4:**
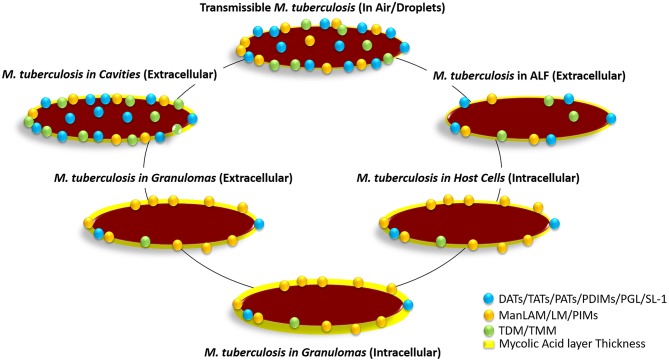
Hypothetical distribution of glycolipids on the *M. tuberculosis* bacterium cell envelope surface at the different stages of infection. The transmissible bacterium is thought to contain a large number of glycolipids (DAT, TAT, PAT, SL-1, TMM, TDM, PDIMS, PIMs, etc.) on the cell envelope driving surface hydrophobicity, which in turn favors its transmission through the air. Upon infection and after reaching the alveolar space, *M. tuberculosis* gets in contact with alveolar lining fluid (ALF; hypophase of the lung mucosa). ALF contains an array of homeostatic hydrolases that significantly alter the *M. tuberculosis* cell envelope surface removing glycolipids and lipoglycans, and thus somehow altering its cell surface hydrophobicity and determining how the bacterium will interact with alveolar host cells. After phagocytosis by AMs, the bacterium is shown to hyperproduce PIMs, as well as switching to metabolic networks such as beta-oxidation, glyoxylate shunt, and the reverse methylcitrate cycle. This metabolic switch within host cells allows the bacterium to break down own and host captured long-chain fatty acids and cholesterol to generate acetyl-CoA and propionyl-CoA, increasing its production of mycolic acids in detriment of producing glycolipids. This bacterium metabolic state is thought to be maintained at latency stages when the bacterium remains intracellular within granulomas. Upon reactivation, the bacterium metabolic state changes again increasing glycolipid production in detriment of producing mycolic acids, restoring the bacterium surface hydrophobicity, while remaining extracellular within granulomas. This bacterial surface hydrophobicity gets further accentuated when *M. tuberculosis* escapes disrupted granulomas becoming extracellular within cavities, where host-driven hydrolytic destruction of tissues enhances *M. tuberculosis* escape to the blood stream and airways becoming transmissible again closing the cycle. Relative number of molecules and size are not accurately depicted reflecting published experimental data.

The interest of studying the tubercle bacillus lipoids remotes to more than 90 years ago by Anderson et al. in 1929 stating: “*The crude wax obtained from the chloroform extract of the human type of tubercle bacillus, Strain H-37, yielded on purification, two fractions; viz., a white solid substance designated as purified wax and a soft yellowish brown salve-like mass which was called soft wax and it possessed a slight but agreeable perfume-like odor*” (20). Since then, a fascinating diversity of lipid structures and their biological activities have been and are still being described. In this context, glycolipids are one of the major *M. tuberculosis* cell envelope constituents, defined by their polarity, cytotoxicity, and/or immunological properties (17) (Figure 1). These mainly comprise the acyl trehaloses family sharing a common α-D-Glc*p*(1→ 1′)-α-D-Glc*p* unit and are the class of *M. tuberculosis* lipids most extensively studied by lipidologists and mycobacteriologists. These specifically include di-, tri-, and penta-acyltrehaloses (DATs/TATs/PATs); mono- and di-mycolyl trehaloses (TMM/TDM); and tetraacyl-trehalose sulfo(glyco)lipids (SLs) (21), and the lesser studied to date, oligosaccharides containing lipids [lipooligosaccharides (LOSs)] (Figure 3). Another class of *M. tuberculosis* glycolipids of important relevance is the phosphatidyl-*myo*-inositol mannose derivatives (PIMs). Some of them are precursors of two major lipoglycans on the *M. tuberculosis* cell surface, the lipomannan, and the mannose capped lipoarabinomannan (ManLAM) (Figure 3). Lastly, and no less important, some *M. tuberculosis* strains also contain phenolic glycolipids (PGLs) (Figure 3). All these glycolipids are thought to perform filler roles in completing the outer leaflet of the asymmetric peripheral lipid layer on the *M. tuberculosis* cell envelope (17). The importance of these lipids in *M. tuberculosis* infection and pathogenesis is described in the sections below and summarized in Table 1 and Figure 4.

**Table 1:** *M. tuberculosis* cell wall glycolipids and lipoglycans and their interaction with the host.

***M. tuberculosis* cell envelope lipid**	**Host cell receptor(s)**	**Phagosome maturation arrest**	**Host immune response**	**Sero** ** activity**	**Cyto** ** toxicity**	**Pathobiological context**	**Disease stage**
Diacyl-, triacyl-, and pentacyl-trehalose (DAT, TAT, and PAT)	ND	Yes	Suppressor (anti-Inflammatory)	Yes	No	Host cell recognition and activation Inhibitor (Mϕ, Nϕ, AT) Phagocytosis inhibitor B-cell priming T-cell proliferation inhibitor	Initial infection establishment Increase transmissibility
Trehalose dimycolate (TDM)	Mincle-FcγR TLRs	Yes	Stimulator (pro-inflammatory)	Yes	Yes	Host cell recognition and activation (Mϕ, Nϕ, AT) Inflammasome activation Apoptosis	Initial infection establishment Granuloma formation and maintenance Cavitation Increase transmissibility
Trehalose monomycolate (TMM)	ND	ND	Stimulator (pro-inflammatory)	Yes	Yes	Apoptosis	Initial infection establishment Granuloma formationand maintenance Cavitation Increase transmissibility
Lipooligosaccharides (LOSs)	ND	ND	Suppressor (anti-inflammatory)	Yes	ND	Host cell recognition (Mϕ, others?) Motility	Initial infection Decrease transmissibility
Sulfated trehalose glycolipid family(SL-1, Ac_2_SGL)	ND	Yes (by SL-1)	Suppressor (anti-inflammatory)	Yes	Yes (Ac_2_SGL only)	Host cell recognition and activation Inhibitor (Mϕ, DCs, others?) Phagocytosis inhibitor Inflammasome inhibition T-cell proliferation activator	Initial infectionestablishment Increase transmissibility
Phenolic glycolipid(PGL-TB)	CR3? TLRs	ND	Anti-/Pro-Inflammatory	Yes	Yes	Host cell recognition and activation (Mϕ, others?)	Initial infection Granuloma formation and maintenance (chronic infection)
Phthiocerol dimycocerosate (PDIM)	Direct insertion into host Mbrs	Yes	Suppressor (anti-inflammatory)	Unknown	Yes	Host cell recognition and activation (Mϕ, DCs, others?)	Initial infection Cavitation Increase transmissibility
Lower-order phosphatidyl-*myo*-inositol mannosides (Ac_x_PIM_1−4_)	CR3, TLRs, DC-SIGN	No	Stimulator (pro-inflammatory)	Yes	ND	Host cell recognition and activation (Mϕ, Nϕ, others?) Early endosome fusion stimulator Oxidative response stimulator Activate T-cell proliferation via CD1	Initial infection establishment
Higher-order phosphatidyl-*myo*-inositol mannosides (Ac_x_PIM_5−6_)	MR, DC-SIGN	Yes (through the MR only)	Suppressor (anti-inflammatory)	Yes	ND	Host cell recognition and activation (Mϕ, Nϕ, others?) Apoptosis modulator Activate T-cell proliferationvia CD1	Initial infection establishment Granuloma formation and maintenance (chronic infection)
Lipomannan (LM)	TLRs, DC-SIGN	No	Stimulator (pro-inflammatory)	Yes	ND	Host cell recognition and activation (Mϕ, Nϕ, others?) Apoptosis modulator Activate T-cell proliferationvia CD1	Initial infection establishment
Mannose-capped lipoarabinomannan (ManLAM)	MR, DC-SIGN	Yes (through the MR only)	Suppressor (anti-inflammatory)	Yes	ND	Host cell recognition and activation (Mϕ, Nϕ, others?) Apoptosis modulator Activate T-cell proliferationvia CD1	Initial infectionestablishment Granuloma formation and maintenance (chronic infection)

## The DAT/TAT/PAT Glycolipid Family

Diacyl- (DAT; a 2, 3-diacyltrehalose), triacyl-trehalose (TAT; a 2, 3, 6-triacyltrehalose), and pentaacyl-trehalose (PAT; a 2, 3, 6, 2′, 4′-pentaacyltrehalose) contain polymethylated acyl groups (22). Recent studies unveiled that DAT family alone isolated from the *M. tuberculosis* cell envelope contains more than 30 molecular species, with each species containing up to six isomeric structures (23).

Their purpose and function in the *M. tuberculosis* cell envelope are still unclear. Some studies related DAT functions to its capacity to negatively regulate the host pro-inflammatory response induced by *M. tuberculosis* during infection in a dose-dependent manner (24). Indeed, DATs do not seem to participate in the receptor-mediated phagocytosis process of *M. tuberculosis*, do not induce host cytotoxicity, but trigger IL-10 production and are capable of inhibiting T-cell proliferation (25), supporting their role in potentially being used by *M. tuberculosis* to offset the host immune response during initial stages of the infection (24) (Table 1). Moreover, other studies indicate the capacity of DATs acting together with PAT (the major polyacyltrehalose of *M. tuberculosis* containing four mycolipenoyl and one fully saturated palmitoyl) in the maintenance of the *M. tuberculosis* cell envelope, as well as in pathogenesis promoting the *M. tuberculosis* survival within host cells (26) and being antigenic (27). In this context, several studies emphasize the potential primary role of DATs in regulating the composition of the *M. tuberculosis* cell envelope, serving as anchors of the *M. tuberculosis* outer material, which blocks *M. tuberculosis* recognition and phagocytosis by host cells (28, 29). In this regard, *M. tuberculosis* DAT mutants have altered cell envelope surface and have enhanced phagocytosis by host cells, but their absence from the *M. tuberculosis* cell envelope does not alter the bacterium capacity to replicate and survive *in vivo* using the mouse model (28, 30). Conversely, other studies have shown that *M. tuberculosis* mutants lacking DATs and sulfoglycolipids cannot block phagosome maturation, thus revealing the importance of these molecules in the *M. tuberculosis* pathogenesis (31). Currently, the contribution of *M. tuberculosis* acytrehaloses to pathogenesis is still understudied, and the main use of these *M. tuberculosis* glycolipids is still for serodiagnosis (32–36).

## The Mycolic Acid Esters of Trehalose Glycolipid Family

Other trehalose-containing family of lipids characterized by their long acyl groups is the mycolyl-containing trehalose lipids (37). These are widely distributed and considered the most abundant, non-covalently associated *M. tuberculosis* cell envelope lipids, providing *M. tuberculosis* with a waxy, impermeable appearance. Both TDM (6,6′-dimycoloyl-α-D-trehalose) and TMM (6-monomycoloyl-α-D-trehalose) have their mycolates oriented toward the cell plasma membrane, presenting their trehalose moiety to the external environment.

TDM (or cord factor) was first obtained after a petroleum ether extraction from cells of a virulent strain of *M. tuberculosis* (38). After the extraction, *M. tuberculosis* presented a drastic disorganization of the typical cellular cords formed in a culture, and the resulting petroleum ether organic extract was toxic in mice. This toxicity was later shown to be related to TDM (39). Subsequently, further studies indicated that TDM toxicity is linked to the regulation of tissue-specific nicotinamide adenine dinuclease (NAD) activity by TDM-induced NADse, decreasing tissue-specific NAD levels by blocking the electron flow along the mitochondrial respiratory chain and thus oxidative phosphorylation (40–44). *M. tuberculosis* TDM has two mycolic acids (or mycolates). Each TDM mycolate has two acyl-chains, a long β-hydroxy chain consisting of 50 to 60 carbons, and a shorter α-alkyl chain typically with 24 carbons (45). Thus, each mycolic acid of TDM is reported to contain between 74 and 90 carbons (45–47). The length and ester-linkages of the mycolates on TDM molecules define their toxicity; a longer mycolate shows higher toxicity (48, 49). Related to the *M. tuberculosis* cell envelope, TDM seems to interlock with ManLAM, which is also found on the surface of *M. tuberculosis*, to form an asymmetrical bilayer and thus directly participates in the maintenance of the *M. tuberculosis* cell envelope integrity (37). Studies removing TDM from the bacterium surface further indicated the importance of TDM in the *M. tuberculosis*-host relationship that takes place in infections *in vitro* and *in vivo* (50, 51). In this regard, *M. tuberculosis* is shown to produce large quantities of TDM during infection and when inside of the phagosome (52). TDM also seems to interlock host fibrinogen, the latter then acting as a cofactor for TDM biological effects (53).

*M. tuberculosis* TDM's biological properties have been described to rely on the whole molecule (54, 55), and these include (Table 1): interacting with Mincle (macrophage-inducible C-type lectin) (56) on the macrophage surface, driving *M. tuberculosis*-host recognition, and interacting with Mincle working together with the Fcγ-receptor transmembrane segment inducing Th1 (e.g., TNF, IL-12), Th2 (IL-6), and inflammasome (IL-1β) pro-inflammation mediators (57–59); actively participating in driving mycobacterial phagosome maturation arrest (55, 60) after phagocytosis of virulent strains of *M. tuberculosis*, allowing the bacillus to survive within the phagocyte; indirectly participating via its recognition by Mincle in the influx of neutrophils to the infection site (61); inducing host cell apoptosis (62–64); participating in the induction and vascularization of lung granulomas (65); being antigenic and having antitumoral activity (63, 66); having adjuvant properties being able to generate optimal antibody responses (67); inducing host cachexia, a typical symptom in active TB disease (68); and being able to trigger non-specific host immune responses against both parasitic and bacterial infections (63, 69–72). TDM is also shown to undergo cyclopropane modifications on its mycolates (73), driving a TDM-dependent reprogramming of the macrophage global gene expression during infection *in vitro* and *in vivo* (74).

TMM is known to be an essential precursor for the biosynthesis of TDM and the mycolic acid layer covalently linked to the arabinogalactan-peptidoglycan (mAGP) core of the *M. tuberculosis* cell envelope (75). Although TDM has been largely studied in its close relation with *M. tuberculosis* pathogenesis, this is not the case for TMM. TMM is thought to be involved in the maintenance of the structure and function of the *M. tuberculosis* cell envelope, working together with arabinogalactan mycolate, TDM (76), or phosphatidyl-ethanolamine, the latter shown to participate in the TMM translocation from the plasma membrane to *M. tuberculosis* cell envelope core (77). In this context, TMM is shown to be used by *M. tuberculosis* to transfer mycolic acids toward molecules like the envelope-linked arabinogalactan. In agreement with this fact, the known secreted immunogenic Ag 85 complex has been identified as a trehalose mycolyltransferase (78, 79).

As TDM, TMM has similar biological properties, also inducing lethal host cytotoxicity and host cell apoptosis; has granulomagenic activity; has adjuvant activity; and is capable of stimulating TNF via activation of the protein kinase C pathway (10, 80–84) (Table 1).

## The Los Glycolipid Family

Another family of trehalose-based lipids present on the *M. tuberculosis* cell envelope is the LOSs. LOSs in the *M. tuberculosis* complex are understudied, produced by some strains (e.g., *M. canettii*) (85), and absent in most clinical isolates of *M. tuberculosis* as well as laboratory strains such as H_37_R_v_, H_37_R_a_, Erdman (86). The majority of their biological attributes are derived from studies performed in other mycobacterial species such as *Mycobacterium kansasii, Mycobacterium smegmatis*, and *Mycobacterium marinum* among others (85–89). LOSs are considered to be located on the surface of the mycobacterial cell envelope (90–92), but their contribution in shaping it is still unclear (93). However, the typical smooth and glossy colony formation observed in the *M. tuberculosis* complex strain *canettii* is speculated to be directly linked to the heavy presence of LOSs in this strain cell envelope (85, 94, 95).

All described LOSs share one trehalose with at least one or two long poly-methylated branched fatty acid chains that can be saturated or unsaturated, and located in both glucose residues of the trehalose end of the polymer (90). Depending on the mycobacterial species, a monosaccharide, or more frequently, an oligosaccharide (2 to 6 carbohydrate residues) is also present linked either at C3, C4, or C6 of the trehalose end (89, 96, 97). LOSs from different mycobacteria present different grade of variability in this oligosaccharide, the most frequent one being a tetraglucose structure [D-Glc*p*(β1→ 3)-D-Glc*p*-(β1→ 4)-**D-Glc*p*-(α1→ 1α)-D-Glc*p***], which includes the trehalose (in bold). Pyruvic acid residues (carboxyethylidene) have also been described giving an anionic character to these molecules.

Related to LOSs biological properties, these may play an important role in regulating bacterial transmission, motility, and biofilm formation; are immunogenic and also phage receptors; and may play a role in the establishment of the infection (86, 98) (Table 1). LOSs are mainly used for serotyping, as LOS-specific antibodies are raised against the immunodominant LOS oligosaccharide. LOSs are also shown to directly or indirectly participate in the mycobacterial infection of host macrophages (86). In this regard, their role in *M. tuberculosis* virulence is unclear. Some studies suggest LOSs being attenuated/masking factors such as DATs, where mycobacterial species containing LOS are readily cleared *in vivo*; conversely, mycobacterial strains lacking LOSs cause chronic and systemic infections *in vivo* (92, 99).

One role of LOSs is to participate in reducing the hydrophobicity of the mycobacterial cell envelope. Studies suggest that *M. tuberculosis* complex strain *canettii* is an ancestor strain, mainly isolated in the horn of Africa, containing abundant LOSs (100). However, as evolution goes, modern *M. tuberculosis* complex strains became more hydrophobic, replacing LOSs in their cell envelope in detriment of adding more apolar lipids. This change of polarity/hydrophobicity is thought to provide critical advantages to the *M. tuberculosis* complex, increasing their capability for aerosol transmission (101), affecting their virulence and pathogenicity (100, 102).

## The Sulfated Trehalose Glycolipid Family

As in the case of the trehalose containing DATs, TATs, PATs, TDMs, and TMMs, sulfated trehaloses (or sulfolipids, designated by SL) (103) are also present in virulent strains of *M. tuberculosis*, specifically SL-1 (104, 105). Sulfate derivatives are rare in nature, where the degree of sulfation could directly change the binding properties directing extracellular interactions between biomolecules from both, *M. tuberculosis* and host cells (106). Concretely, *M. tuberculosis* SLs consists of a trehalose-2-sulfate disaccharide that can be acylated by two [defining the diacylated sulfoglycolipid or Ac_2_SGL (107)] to four [defining SL-1 (108)] very long (up to C_64_) saturated and unsaturated, highly branched fatty acids (109, 110).

Like TDM, SL-1 [and potentially Ac_2_SGL (107)] is involved in the establishment of the infection. SL-1 seems to induce phagosome maturation arrest in macrophages (111); is cytotoxic *in vitro* (112, 113); inhibits macrophage priming/activation (114, 115), phagocytosis, and the inflammasome (115); induces superoxide release in activated neutrophils and monocytes; and are immunogenic with potential for serodiagnosis (33, 116–118) (Table 1). Specifically for Ac_2_SGL (107), this can stimulate and proliferate CD1b-restricted T cells (119), and stimulate the production of Th1 cytokines by CD8^+^ T cells (119). However, *in vitro* and *in vivo* studies, the latter using different animal models, dispute the role of SL-1 in *M. tuberculosis* virulence and pathogenesis (114–117, 120–122). SL-1 has been shown acting together with TDM, damaging the mitochondrial structures in the host cell *in vitro*; however, SL-1 alone intraperitoneally injected in mice did not induce cytotoxicity. *M. tuberculosis* mutants (*pks2*) lacking both SL-1 and its precursor Ac_2_SGL indicate that both are not required for *M. tuberculosis* to grow and cause pathogenesis *in vivo*. Conversely, an *M. tuberculosis* mutant (*mmpl8*) only lacking SL-1 but accumulating Ac_2_SGL were shown to be attenuated *in vivo*, indicating that the SL-1 precursor Ac_2_SGL is indeed immunogenically participating in *M. tuberculosis* virulence (108). In this regard, it has been suggested that as Ac_2_SGL is a precursor of SL-1 lacking two acyl groups (phthioceranic acid and hydroxyphthioceranic acid), it could be thus plausible that *M. tuberculosis* uses the ratio of SL-1/Ac_2_SGL in its cell envelope to modulate the host immune response to its advantage (119, 123).

## Other Trehalose Glycolipid Family

Lesser-studied *M. tuberculosis* trehalose-containing lipids are the phthienoates (or mycolipenates) and hydroxyphthienoates (or mycolipanolates). These consist of four species with different polarities (124). The less polar species have acyl substituents mainly straight-chain 16:0 and 18:0 acids and 2,4,6-trimethyl tetracos-2-enoic (C27 phthienoic or mycolipenic) acid, or a 3-hydroxy-2,4,6-trimethyltetracosanoic (C27-hydroxyphthienoic or mycolipanolic) acid. The polar pair species mainly have straight-chain 16:0 and 18:0, C27-mycolipanolic acid, minor proportions of C25- and C27-mycolipenic acids, and major proportions of 2,4-dimethyldocosanoic acid (124, 125). Their relative contribution to the *M. tuberculosis* pathogenesis remains uncertain, but their function in maintaining the integrity of the cell envelope is stipulated (124).

## The PGL Family

Other *M. tuberculosis* glycolipids containing oligosaccharides are the PGLs. PGLs are considered a glycosylated form of phthiocerol dimycocerosates (PDIM) (126). These glycolipids have been mainly studied in *Mycobacterium leprae* (triglycosylated PGL-I) (127–129) and *Mycobacterium marinum* (monoglycosylated PGL), being used for diagnostic testing in leprosy patients (130). The exact structure of PGL is species-specific, and its biological properties are starting to be elucidated. In this regard, while in leprosy, *M. leprae* PGL-1 subverts tissue macrophages to produce neurotoxic radical nitrogen species (e.g., nitric oxide) leading to nerve damage (131). Along this line, PGL-1 has also been shown to be effective oxygen radicals scavengers (132). In *M. marinum* infection, PGL drives phagosome maturation arrest (133), and allows the bacterium to escape from hostile tissue-resident macrophages during the initial stages of the infection (131, 134, 135). By doing so, *M. marinum* uses PGL to infect resident macrophages and induce the secretion of the chemokine (C-C motif) ligand 2 (CCL2) attracting non-bactericidal monocytes, which will be subsequently infected by *M. marinum* and used as Trojan horses to cause a systemic infection (134–136).

As LOSs, PGLs are not present in all *M. tuberculosis* complex strains. These have been found in abundance in the *M. tuberculosis* complex *canettii* strain (PGL-Tb) (137) and in same *M. tuberculosis* clinical isolates of the W-Beijing family. Due to the different amounts of PGLs produced by different *M. tuberculosis* strains (138), their use for *M. tuberculosis* serodiagnostic did not prove to be useful as TB patients presented significant variations in their response to this antigen (85). Interestingly, the hypervirulent phenotype observed in several strains of *M. tuberculosis* (e.g., the Beijing strain HN878) has been associated with the presence of PGL and triglycerides in their cell envelope (139, 140).

Contrary to the debatable PGL hypervirulence effects where *M. tuberculosis* PGL-deficient strains induce pro-inflammation (141), recent studies, using a necrotic *M. tuberculosis* mouse model, show that loss of *M. tuberculosis* PGLs decreases the Th17 response (e.g., IL-17A), where IL-17A production seems critical to limit the development of hypoxic necrotic granulomas and reduces disease severity in TB (142). Thus, indirectly, *M. tuberculosis* PGL may favor *M. tuberculosis* infection reducing tissue damage. This concept is supported by other studies analyzing multiple clinical isolates, where the clinical spectrum of *M. tuberculosis* infection resulting in active TB or latent infection could be dictated not only by the host but also by the amounts and ratios of surface-exposed glycolipids defined by the strain genotype (134, 143, 144). Thus, serum opsonized *M. tuberculosis* clinical isolates expressing abundant PGL on their surface had reduced phagocytosis by macrophages, but the ingested *M. tuberculosis* bacilli replicated at faster rate (144). This was hypothetically attributed to the opsonization of *M. tuberculosis* surface-exposed PGL by host-soluble complement component 3 (C3) and the subsequent use by *M. tuberculosis* of the macrophage phagocytic complement receptor 3 (CR3) to infect host cells (144). Later studies confirmed that the sugar moiety of PGL is unique in its capacity to bind the lectin domain of CR3 for efficient infection of human macrophages, and *M. tuberculosis* PGLs are able to inhibit Toll-like receptor 2-triggered NF-κB activation and thus the pro-inflammatory response, thus assisting *M. tuberculosis* to subvert the host immune response (145) (Table 1). This capacity to dampen the host response is further corroborated by studies using *M. tuberculosis* PGL analogs, where these inhibited the production of Th1 (TNF), Th2 (IL-6), and the inflammasome (IL1-β)-related responses in a Toll-like receptor 2-mediated manner (143). Other supporting studies using recombinant BCG expressing PGL-1 show that this strain can exploit the host CR3, promoting bacterial uptake but at the same time inhibiting pro-inflammatory responses (e.g., TNF) (146). Overall, the direct interaction of PGL with host cell receptors is still unclear. Studies focused in determining which host receptors could recognize and bind *M. tuberculosis* complex BSA-conjugated glycans indicate that dendritic cell-specific ICAM-3-grabbing non-integrin (DC-SIGN or CD209), the DC-SIGN homolog DC-SIGNR (for DC-SIGN related), Langerin (CD207, C-type lectin receptor on Langerhans cells), the mannose receptor (MR or CD206), galactose receptor, dendritic cell C-type lectin 2 (Dectin-2), macrophage-inducible Ca^2+^-dependent lectin receptor (Mincle or CLEC4E), and BDCA-2 (CLEC4C or CD303) had low affinity for the rhamnose (6-deoxy-L-mannose) and fucose (6-deoxy-L-galactose) found in PGLs, indicating that PGL could be targeted by other, yet to be discovered, host C-type lectins (147).

Interestingly, studies also highlighted the role of PGL in BCG efficacy, where a mutant BCG (deleting *fadD28*) lacking both PDIMs and PGLs not only made BCG more attenuated in mice but also reduced its efficacy as a vaccine against *M. tuberculosis* infection (148).

## The Mannose-Containing Glycolipid Family

This family involves phosphatidyl-*myo*-inositol (PI) glycosylated derivatives known as PIMs and the predominant mannosyl-β-1-phosphomycoketides (MPM). PIMs are important lipids in the cell envelope of *M. tuberculosis*, both as key cell envelope constituents involved in metabolic processes and as essential participants in *M. tuberculosis*-host interactions. PI is an acidic (anionic) phospholipid consisting of a phosphatidic acid linked via a phosphate group to *myo*-D-inositol. PIMs are considered, together with phosphatidyl-ethanolamine, the major phospholipid components of the *M. tuberculosis* cell envelope (75). There are found on the *M. tuberculosis* cell envelope as a mixture of compounds differing from each other by their number of mannose and fatty acid residues. The basic structure of PIM_1_ consists of a mannosyl unit attached to position C-2 of the *myo*-inositol of a PI anchor. Position C-6 of *myo*-inositol can be further substituted by an α-D-mannosyl or a linked di- or trimannosyl unit, giving PIM_2_, PIM_3_, and PIM_4_, respectively. The terminal mannose residue of PIM_4_ can be further substituted at position C-2 by a α-D-mannosyl leading to PIM_5_, which can also be further substituted at the same position leading to PIM_6_, the higher PIM encountered in mycobacteria to date (10). PIMs are shown to be located both in the *M. tuberculosis* plasma membrane and on the cell envelope surface (149). PIMs are intrinsically highly heterogeneous not only due to their mannose content but also due to the number and nature of their acyl groups. PIMs can be mono- to tetra-acylated, where the basic PIM is the diacylated form (150) esterified by palmitic (C16) and tuberculostearic (TBST or 10-methyl-octadecanoic) acids on C-1 and C-2 position of the glycerol, with a third fatty acyl substituent on the C-6 position of the (1→ 2) linked mannose to the inositol and with a fourth fatty acyl on the C-3 of the *myo*-inositol (150, 151). To simplify, PIMs can be grouped into lower- and higher-order PIMs depending on their number of mannoses, where lower-order PIMs contain one to four mannoses and higher-order PIMs contain five to six mannoses (152). The most common PIMs found in *M. tuberculosis* complex are AcPIM_2_ and Ac_1_PIM_2_ (di- and triacylated PIM_2_) and AcPIM_6_ and Ac_1_PIM_6_ (di-and triacylated PIM_6_) (150). All lower-order PIMs have a terminal α(1→ 6)-mannose, are exposed on the cell surface, and directly participate in the establishment of the infection, playing a role in macrophage recognition and phagocytosis process through association with the non-opsonic domain of CR3 (153). Lower-order PIMs also participate in facilitating trafficking processes within the phagocyte by promoting early endosomal fusion with *M. tuberculosis* containing phagosomes, thus providing nutrients to the bacterium (154) (Table 1).

In contrast, all higher-order PIMs have a terminal α(1→ 2)-mannose. In the case of Ac_x_PIM_5_, it is a single α(1→ 2)-mannose, and in the case of Ac_x_PIM_6_, it is an α(1→ 2)-dimannoside similar to the mannose caps of mannose-capped lipoarabinomannan [ManLAM, reviewed in Turner and Torrelles (155)]. Only tri-acylated forms of higher-order PIMs are shown to interact with the MR and interfering with trafficking pathways resulting in phagosome maturation arrest (152). Conversely, all *M. tuberculosis* PIMs seem to interact with DC-SIGN (152), although differences in DC-SIGN PIM recognition specificity may be species-dependent (156). Further studies have also shown that cytosolic soluble CD1e is involved in AcPIM_6_ processing and presentation via CD1 with subsequent T-cell activation (157) (Table 1).

Overall, without discerning the type of PIMs used, these have been shown to participate in host cell recognition, phagocytosis, oxidant and cytokine production, regulating intracellular vesicular trafficking, regulating signaling pathways, apoptosis, and antigen presentation (158–160) (Table 1). However, as pointed out, these intrinsic PIM properties, apart of being species-specific and depending on their heterogeneous nature, also may be derived from differences in the protocols used for PIM isolation and for *in vitro* experiments including immune cell types and procedures to generate them (160, 161).

To date, the role and metabolism of PIMs during *M. tuberculosis* infection and pathogenesis is still unclear, as different PIMs are recognized by different receptors triggering different host responses and outcomes. PIMs are downregulated in stationary phase (162), but it has been observed that the production of PIMs by *M. tuberculosis* is enhanced during infection in primary human macrophages (163). The latter result indicates that *M. tuberculosis* can adapt to the environment during infection by modulating its cell envelope composition (164–168). That PIM expression levels during early stages of infection may be in part directly or indirectly involved in bacterial growth, virulence, and entry into persistence is still unclear; however, studies show that a PIM-hyperproducing *M. tuberculosis* strain (ΔRv2623) is hypervirulent *in vivo* being unable to establish a chronic infection (163), while a PIM-hypoproducing *M. tuberculosis* strain (ΔRv1747) is attenuated *in vivo* in mice (169) (Table 1).

Another *M. tuberculosis* mannose-containing glycolipid is MPM. This glycolipid consists of a mannosyl-β-1–phosphoisoprenoid with a C32 4,8,12,16,20-pentamethylpentacosyl chain (170) and has been shown to activate CD1c-restricted T cells (171).

## The Role of Cell Envelope Glycolipids In *M. tuberculosis* Evolution, Adaptation To The Host Environments, Drug Resistance, and Pathogenesis

There are no doubts that the cell envelope of *M. tuberculosis* has evolved over the years to become the perfect shield for this pathogen, from the perspective of its hydrophobicity, transmissibility, adaptation to the host, drug resistance, virulence, and pathogenesis.

Several studies have discussed at large how *M. tuberculosis* has evolved increasing the hydrophobicity of its cell envelope to its advantage. In this regard, the role of the cell envelope lipids in the transition of environmental mycobacteria becoming highly transmissible, pathogenic and host-dependent is pointed out (102). The possibility that ancient *M. tuberculosis* complex strains such as *M. canettii*, mainly isolated in the horn of Africa (172, 173), have evolved has been explored, that is, changing their metabolism and increasing their cell envelope hydrophobicity as they evolved to the modern *M. tuberculosis* complex strains, e.g., changing their cell envelope's outmost exposed lipid polarity and thus evolving into a more hydrophobic organism. This was achieved by stopping the production of polar lipids such as LOSs or PGLs (which currently are just described in a subset of *M. tuberculosis* complex modern strains, Beijing clinical isolates), reducing their polarity (e.g., by losing sugars or adding acyl chains in their cell envelope glycolipids, e.g., DAT, TAT, PAT, SLs, TDM, TMM), and increasing the presence of apolar lipids in their cell envelopes [e.g., dimycocerosates of the phthiocerol family (PDIMs)] (102). Thus, cell envelope hydrophobicity could be linked to a simplified cell envelope, efficiency in transmissibility, and increased pathogenesis [extended review in Jankute et al. (102)]. Conversely, other studies shuffle the possibility that modern *M. tuberculosis* complex strains are adapting to the host by decreasing the hydrophobicity of their cell envelopes by adding mannose-containing components on their cell envelope, mimicking mammalian glycoproteins (Figure 2), and thus deceiving the host immune response during infection (10).

Looking at both scenarios and at the properties described for the glycolipids present in the cell envelope of *M. tuberculosis* complex, these two possibilities seem compatible, but potentially with different outcomes. Increasing hydrophobicity allows *M. tuberculosis* to be highly transmissible, and this could be the case for CDC1551, an *M. tuberculosis* strain shown to have this highly transmissible property (174). Increasing the hydrophobicity of *M. tuberculosis* can also trigger increased interactions of *M. tuberculosis* bacilli with signaling receptors that drive pro-inflammation and cellular trafficking to the infection site, and depending on the metabolic status of the host and the bacterium at this initial encounter, could result in active TB disease. This is supported by the role of triglycerides in driving hypervirulence in place of the initially described PGL (139, 140), which seems now to be involved in attenuating the host immune response (142).

Conversely, the other scenario is decreasing hydrophobicity by *M. tuberculosis* adding mannose-containing biomolecules in its cell envelope, such as the glycolipids PIMs, and their biosynthetically related lipoglycans and their derivatives [lipomannan, ManLAM, mannan, and arabinomannan (10)] (Table 1). *M. tuberculosis* complex strains with high mannose content in their cell envelope could be less effectively transmissible; however, when infecting the host, these trigger an anti-inflammatory response by interacting their mannose-containing glycolipids with homeostatic receptors of phagocytes [e.g., the MR (10, 175)], confusing the host cell. This scenario can result in a permissive intracellular environment for *M. tuberculosis* to permanently stay, without being detected, allowing host cells to control the infection, ultimately driving to the latency state. This is supported by the role of other glycolipids present in modern *M. tuberculosis* complex strains, such as DATs, which are shown dampening the host immune response (24).

The fact is that *M. tuberculosis*-host cell interactions, infection, and potential outcomes may also depend on the *M. tuberculosis* metabolic status prior to and during infection (Figure 4). *M. tuberculosis* overproduces PIMs during *in vitro* infections in macrophages (163). *M. tuberculosis* Ac_1_PIM_6_, as well as ManLAM, interacts with the MR defining a pathway of intracellular survival for *M. tuberculosis* and at the same time dampens the host immune response potentially benefiting *M. tuberculosis* adaption to the host (152, 176). However, a third unexplored possibility is that the cell envelope of *M. tuberculosis* shaping directly depends on the host lung environment, during the early stages of infection (in the alveolar space) or in later stages of infection, in cavities, when transmissible bacilli are shaped to be transmitted. In both cases, *M. tuberculosis* remains extracellular. In the initial stages, when infection occurs, *M. tuberculosis* is deposited on the alveolar space of the lung, where it will find the human lung mucosa before encountering any host cell [e.g., AMs, alveolar epithelial cells (7)]. In this scenario, the alveolar epithelium is covered by lung mucosal fluid, which is composed of two phases, a lipid layer called surfactant (mainly composed of phospholipids) and an hypophase called alveolar lining fluid (or ALF). Host cells are thought to be immersed and operating in the ALF. ALF is thought to trap any particle that reaches the alveolar space. ALF has been described to contain an array of hydrolytic enzymes [hydrolases (164)], whose main function is to assist host cells in recycling ALF every 18 h (177). Studies have demonstrated that the nature of these host hydrolases impacts the *M. tuberculosis* cell envelope as it has been described, where 15-min exposure of *M. tuberculosis* to human ALF reduces the cell surface presence of ManLAM and TDM by ~70 and ~40%, respectively (164). Other affected lipids significantly reduced from the *M. tuberculosis* cell envelope upon contact with human ALF are triglycerides, PDIMs, SLs, PIMs, and at lower degree DATs (personal communication). The impact of these human ALF modifications on the *M. tuberculosis* cell envelope results in a better control of the infection by human phagocytes (macrophages and neutrophils) by increasing *M. tuberculosis*-containing phagosome-lysosome fusion and acidification events (164, 178). Thus, the repertoire of lipids exposed on the *M. tuberculosis* cell envelope surface during the infection initial stages and prior to the first contact with host cells may in fact depend on the host ALF status, e.g., activity of the ALF hydrolases shaping the cell envelope.

Nevertheless, these *M. tuberculosis* cell envelope glycolipid modifications also result in the release of *M. tuberculosis* cell envelope fragments to the milieu (164). Their implications in *M. tuberculosis* pathogenesis are starting to be elucidated, but studies have shown that these fragments further assist macrophages in controlling *M. tuberculosis* infection by further increasing phagosome-lysosome fusion events in an IL-10-dependent manner (179), as well as drive neutrophils to control *M. tuberculosis* infection better by increasing their oxidative response (180).

The fact is that the status of the host lung environment could somehow “*normalize the cell envelope of M. tuberculosis*,” as well as redirecting *M. tuberculosis* adaptation to the host. Furthermore, *M. tuberculosis* exposure to ALF alters the cell envelope but also differentially drives how other innate soluble components described in the lung mucosa will interact with *M. tuberculosis* and drive infection and potentially disease outcome. In this context, TB comorbidities such as aging (181, 182), HIV, diabetes, smoking, etc., may impact the status of ALF, its hydrolases, and other soluble innate components functionality (182), and thus, the *M. tuberculosis* cell envelope may be differentially impacted depending on the status of the human ALF at the moment of the establishment of the infection.

This scenario has been tested in studies where the BCG vaccine has been exposed to human ALF and subsequently used to vaccinate mice showing that ALF-exposed BCG has an altered cell envelope and increased protection against *M. tuberculosis* challenge (183). Based on this study where ALF modified the BCG cell envelope and drove improved protection, studies delipidating BCG mostly removing apolar lipids showed the ability of this delipidated BCG to be delivered directly into the lung, minimizing tissue inflammation and to further increase protection against *M. tuberculosis* infection and significantly reducing tissue damage, the hallmark of TB disease (184). All these studies put in trial the role of the *M. tuberculosis* glycolipids during infection, how *M. tuberculosis* metabolically adapts to the lung environment, how *M. tuberculosis* uses its cell envelope glycolipids to mask its presence to host cells or to confuse them by mimicking mammalian structures, and how *M. tuberculosis* reconstitutes and reorganizes its cell envelope during infection and in the different environments that may encounter within the host. These environments could be extracellular at the earlier stages of infection, after escaping the infected cell via necrosis, or in cavities, where hypothetically *M. tuberculosis* bacilli could be exposed to lung tissue hydrolases causative of cavities, and thus, these hydrolases could shape bacteria that escape to the airways and are ready to be transmitted. These environments could also be intracellular within host cells in the alveolar space (e.g., AMs and alveolar epithelial cells) or within the interstitium in granuloma structures.

When it comes to drug resistance, limited information exists about the exact glycolipid composition and their role conferring the drug resistance phenotype in multi- (MDR), extensive- (XDR), and extreme- (XXDR) drug-resistant *M. tuberculosis* strains. In this regard, studies investigating *M. tuberculosis* strains containing rifampicin drug resistance-related mutations show that these strains change their cell envelope lipid composition by overexpressing PGL biosynthesis structurally related PDIMs influencing the macrophage metabolism and response during infection (16). Other studies looking at the role of tesA (a type II thioesterase) in *M. marinum* indicated a potential link between increased drug susceptibility and the lack of PDIM and PGLs on the bacterium cell envelope (185). Additional studies found that mycolic acid cyclopropanation is essential for *M. tuberculosis* viability, drug resistance, and cell envelope integrity (186). Overall, how glycolipids participate in the development of drug resistance directly or indirectly rearranging the *M. tuberculosis* cell envelope is still uncertain. Studies performed using transmission electron and atomic force microscopy techniques dig into this question showing that MDR-, XDR-, and XXDR-*M. tuberculosis* strains have thicker cell envelope and irregular cell surface with tubular extensions than susceptible strains (187, 188). As some XDR- and XXDR-*M. tuberculosis* strains are related to the Beijing family (189), which are shown to have their cell envelope overpopulated with triglycerides, it is plausible to question any relationship between the abundance of a specific hydrophobic lipid on the *M. tuberculosis* cell envelope and drug resistance (190). Many of these questions remain unanswered.

At the end, studies have been focused on depicting the role of purified glycolipids on *M. tuberculosis*-host cell interactions and pathogenesis, using purified lipids, embedded into vehicles (coated in beads or nanoparticles), or using *M. tuberculosis* glycolipid(s)-depleted mutants. This conferred us an enormous knowledge about their role in *M. tuberculosis* cell envelope rearrangements, adaptation, metabolism, catabolism, virulence, and pathogenesis. However, it is still unknown how *M. tuberculosis* uses these lipids during the different stages of infection, how the host shapes and alters them, and how the status of the host with active TB and *M. tuberculosis* itself works together within the cavities to define the successful transmissible form of *M. tuberculosis*.

In this context, persistence of *M. tuberculosis* within host cells and in granulomas depends on the bacterial use of lipid metabolic networks such as beta-oxidation, glyoxylate shunt, and the reverse methylcitrate cycle, to break down own and host captured long-chain fatty acids and cholesterol to generate acetyl-CoA and propionyl-CoA (191, 192). *M. tuberculosis* could regulate acetyl-CoA and propionyl-CoA production depending of the status of the host. Where during initial infection stages and granuloma establishment and maintenance (chronic infection), *M. tuberculosis* converts acetyl-CoA into mycolic acids to thicken its cell envelope and to use them as energy storage units in detriment of producing SL-1, DAT, and PAT, among others (47, 193, 194). Conversely, during reactivation leading to cavity formation, *M. tuberculosis* could further convert acetyl-CoA into mycolic acids, further increasing their production (which in turn are used to produce TDM and TMM), and convert propionyl-CoA and methyl malonyl-CoA into Ac_2_SG, SL-1, PAT, and PDIM (methyl-CoA-lipids) (14, 195, 196), increasing its cell envelope hydrophobicity, which will enhance its host to host transmissibility (Table 1, Figure 4).

## The Role of *M. tuberculosis* Cell Envelope Glycolipids in Vaccination Strategies

*In vitro* and *in vivo* studies using mycobacterial lipids and lipoglycans have shown that these induce both humoral and CD1-restricted T cell-mediated responses in the context of BCG vaccination (197–200). In this context, the CD1 locus encodes five related proteins (CD1a–e). CD1a–c are shown to present antigens to T cells. Concretely, CD1a is shown to present lipopeptides (201); CD1b presents mycobacterial cell envelope lipids and lipoglycans, such as mycolic acids, glucose monomycolate, glycerol monomycolate, SLs, LAM, and PIMs, among others (119, 202–205); and CD1c presents isoprenoid phosphoglycolipids (206). CD1d presents antigens to NKT cells, with the sponge-derived α-galactosyl-ceramide being the major antigen described (207). CD1e is located intracellularly and shown to present metabolized PIM_6_ (157). Overall these data indicate that mycobacterial lipids could directly participate in generating long-standing protective immune responses via CD1-restricted T cells during *M. tuberculosis* infection. The role of these lipid-restricted CD1 T-cell populations in generating a protective immune response against *M. tuberculosis* infection has been debatable due to the lack of human homologs of CD1a, CD1b, and CD1c in mice, but studies using human CD1a–c transgenic (hCD1Tg) mice (208) concluded that, contrary to what has been observed in NK T cells, CD1-restricted T cells in reality exhibit delayed primary responses and more rapid secondary responses, similar to conventional T cells, indicating that these lipid-restricted CD1 T-cell populations in reality participate in adaptive immune responses upon mycobacterial infection and thus embrace the use of mycobacterial lipids to target these CD1-restricted T cells for vaccine strategies.

Conversely, for several years, the use of adjuvants in TB vaccination strategies relied on liposomes and oil extracts (209–211). Current vaccine strategies evolved the adjuvant field by targeting the stimulation of pattern recognition receptors, such as Toll-like receptors (212). These new generations of adjuvants include cationic lipids, micro-/nanoparticles, cytokines, toxin derivatives, CpG-containing DNA-based molecules, antimicrobial peptides/proteins, and mycobacterial proteins conjugates and lipids (209). These adjuvants in combination with the current BCG vaccine or with vaccine subunits are shown to be successful in boosting vaccine efficacies (209, 210, 213–215). In this context, there are ongoing vaccination strategies using mycobacterial lipids (or their analogs) as adjuvants driving better and prolonged protection. As an example, the combination of the Hybrid 1(H1) subunit vaccine with the liposomal adjuvant CAF01 [composed of a cationic liposome vehicle (dimethyldioctadecyl-ammonium) and the synthetic TDM analog, trehalose-6,6-dibehenate or TDB] (216–218). This novel vaccine seems to be well tolerated and able of generating long-lasting protective T-cell responses (216).

Having said this, we need to account that *M. tuberculosis* is a master manipulator of host immune responses, being able to passively (directed by the host) or actively (directed by the bacterium itself) change its metabolism and cell envelope lipid composition, adapting to the different adverse host environments (e.g., ALF, AMs, granuloma) during the course of infection (10, 219–222). Thus, mycobacterial lipids may generate or mask a protective host immune response against infection depending on the human lung environment and thus their debatable potential application in vaccine strategies, which has been reviewed by us in detail elsewhere (223, 224).

Overall, a comprehensive study of the *M. tuberculosis* cell envelope lipid constitution during infection could be critical to develop novel vaccine strategies. Our *in vitro* data demonstrate that *M. tuberculosis*, upon contact with the lung mucosa from healthy adult individuals, is temporally depleted of surface exposed lipids resulting in a better control of the infection by alveolar resident cells (164, 178–180, 225). Our *in vivo* data also show that this temporal depletion of lipids on the *M. tuberculosis* surface results in a better control of *M. tuberculosis* growth in the lung of infected mice over time (personal communication). However, this is not observed when *M. tuberculosis* is upon contact with the lung mucosa of healthy elderly individuals (182) or upon contact with the lung mucosa of HIV+ individuals (personal communication). Instead, in both of these cases, *M. tuberculosis* contact with the lung mucosa of the elderly and HIV+ populations accentuates its intracellular growth in AMs (*in vitro*) and in mice (*in vivo*) (182) (personal communication). These results point out the importance of the status of the human lung mucosa in altering the *M. tuberculosis* cell envelope lipid composition (164), resulting in a successful immune response initially protective against *M. tuberculosis* infection and intracellular growth. Subsequently, when inside human macrophages, *M. tuberculosis* seems to overproduce specific lipids, e.g. PIMs (163). Thus, the host-directed temporal removal of lipids and the bacterial overproduction of lipids within host cells may be important to understand which lipids (their presence and/or absence) are important to generate a prolonged and protective immune response, which may improve current vaccination strategies.

## Conclusions

*M. tuberculosis* is proven to expend energy elaborating a great variety of glycolipids of rather unusual structure, both in the inner cell envelope and exposed on its cell surface. These include mainly three groups of glycolipids, trehalose-containing lipids [e.g., acylglucosides (DAT, TAT, PAT), sulfatides (SLs), and LOSs], the 6-deoxy-pyranose containing phenolic glycolipids (e.g., PGLs), and the ubiquitous mannose-containing glycolipids (e.g., PIMs). Some of these glycolipids are described as *M. tuberculosis* virulence factors being recognized by specific host cell receptors (or directly by their interaction with membranes) and thus helping the bacillus to survive within host cells. Therefore, enzymes involved in glycolipids biosynthesis have been and are extensively studied as these may represent potential drug targets (226). In this context, the role of the *M. tuberculosis* cell envelope lipids during dormancy, nutrient starvation, hypoxia, and oxidative stress has been reported using the *in vitro* Wayne's model, pointing out that under these conditions *M. tuberculosis* adapts its proteome and lipidome (222, 227–231).

Attempts to define glycolipids biological function by genetically manipulating *M. tuberculosis* to obtain glycolipid deficient mutants are somehow providing us if these glycolipids are important for *M. tuberculosis* infection and pathogenesis; however, these studies may bypass the impact of *M. tuberculosis* cell envelope rearrangements and the host environment impact on these glycolipids or new rearrangements due to the mutation. Thus, the production of mutants or conditional mutants that provide different levels of production of these glycolipids in the *M. tuberculosis* cell envelope at different stages of the infection may provide us more accurate information about the role of these glycolipids during infection and their real implication in pathogenesis, in place of studying mutants lacking a specific glycolipid.

## Author Contributions

AG-V, JC, and JT made substantial contributions to the conception, writing, and editing of this review.

### Conflict of Interest

The authors declare that the research was conducted in the absence of any commercial or financial relationships that could be construed as a potential conflict of interest.
